# Elucidating the Role of Honey Bees as Biomonitors in Environmental Health Research

**DOI:** 10.3390/insects14110874

**Published:** 2023-11-14

**Authors:** Katharina Sophia Mair, Johanna Irrgeher, Daniela Haluza

**Affiliations:** 1 Department of Pediatrics and Adolescent Medicine, Division of Pediatric Pulmonology, Allergology and Endocrinology, Medical University of Vienna, 1090 Vienna, Austria; 2 Center for Public Health, Department of Environmental Health, Medical University of Vienna, 1090 Vienna, Austria; 3 Department of General, Analytical and Physical Chemistry, Chair of General and Analytical Chemistry, Montanuniversität Leoben, 8700 Leoben, Austria

**Keywords:** honey, *Apis mellifera*, particulate matter, biomonitoring, air pollution

## Abstract

**Simple Summary:**

The increasing release of harmful pollutants into our environment threatens our health. To detect these dangerous substances, scientists are using special detectors called “biomonitors”, and honey bees are some of the most helpful ones. Honey bees collect pollutants from the air, soil, and water when they go out to find food, which makes them exceptional indicators of what is going on in our environment. In our recent study, we looked at how well honey bees can help us monitor pollutants from an environmental health perspective. We searched through scientific databases and found 19 studies on this topic published between 2010 and 2020. Most of these studies looked at heavy metals in honey bees and hive products like honey. The whole honey bee was found to be the most reliable biomonitor. Overall, this research tells us that honey bees can help us monitor pollutants in our environment.

**Abstract:**

Recently, the One Health concept, which recognizes the interconnectedness of environmental, animal, and human health, has gained popularity. To collect data on environmental pollutants potentially harmful to human health over time, researchers often turn to natural organisms known as biomonitors. Honey bees, in particular, prove to be exceptionally valuable biomonitors due to their capacity to accumulate pollutants from the air, soil, and water within a specific radius during their foraging trips. This systematic literature review summarizes the previous application of the bee species *Apis mellifera* in pollutant monitoring in articles published during the period of 2010–2020. Nineteen studies were included in this systematic literature review. Of these studies, the majority (*n* = 15) focused on the detection of heavy metals in honey bees and beehive products, while 4 studies focused on air pollution by polycyclic aromatic hydrocarbons or particulate matter. The matrix most often applied was the whole honey bee. The included studies demonstrated that honey bees and hive products deliver quantitative and qualitative information about specific pollutants. In this regard, the whole honey bee was found to be the most reliable biomonitor. We found that the included studies differed in design and the methods used. Standardized studies could foster a more consistent interpretation of the levels detected in beehive matrices from an environmental health perspective.

## 1. Introduction

The assessment of global pollutants with the aim of regulating and reducing toxic values is a key aspect of environmental health and epidemiological research [1,2]. Various methods are available to gather essential data on environmental pollution presence and levels. Biomonitoring, in particular, has gained popularity in health research due to its efficiency, specificity, and cost-effectiveness [3]. In the realm of environmental pollution monitoring, significant attention has been directed toward the study of honey bees and beehive products. This collective body of research has yielded valuable insights into the utilization of beehive products as effective biomonitors, encompassing a wide spectrum of approaches, methodologies, and practical applications. Honey bees are especially useful in this respect, as they simultaneously assess for various contamination routes during their foraging trips [4]. The earliest publication on the use of honey bees as bioindicators dates back to 1935, when Svoboda reviewed the negative effects of industrial pollutants on honey bees in former Czechoslovakia, as stated by Porrini et al. [5]. Since then, honey bees have been used in numerous biomonitoring studies, ranging from assessing radionuclides after the nuclear plant incident at Chernobyl to determining pesticide contamination at agricultural sites [5,6,7,8,9].

Several characteristics make honey bees and beehive products excellent indicators for environmental contamination. First, honey bees are ubiquitous and easily kept and collected [5,10]. Furthermore, they conduct several foraging trips a day (approximately 12–15 in their active period), covering an area of approximately 7 km^2^ [11,12]. During these foraging trips, honey bees are exposed to numerous contaminants in the environment [13]. Atmospheric residues may settle directly on their hairy bodies, whereas they ingest other pollutants during the gathering of pollen, drinking of nectar, or harvesting of resin for propolis production [13,14]. It is estimated that one honey bee colony (consisting of approximately 20,000 bees) gathers about ten million micro-samples of nectar and pollen per day [5]. Furthermore, the foraging honey bee population collects honeydew, resin, and water. Thus, honey bees gather numerous samples of the surrounding environment, accumulating contaminants from the air, water, and soil [5,15]. Upon their return to the hive, forager bees transport the collected contaminants, introducing them into the hive, where they subsequently accumulate in beehive products like wax and honey [14,16,17]. 

In light of the growing recognition of the interplay between ecological, human, and animal health, the concept of “One Health”, coined by the World Health Organization (WHO), has emerged as a pivotal framework for addressing complex global challenges [18]. Within this multifaceted landscape, honey bees have garnered substantial attention for their crucial role in both environmental ecosystems and the well-being of diverse species, including humans [19]. To gain insight into the current use of honey bees and beehive products in environmental monitoring, we conducted a systematic literature review focusing on the matrix of interest and its ability to detect certain environmental pollutants, the applied methods, as well as the assessed levels of contaminants. The underlying research question was as follows: Can honey bees and beehive products be used as reliable biomonitors in environmental health research to assess the level of contaminants in the environment? The defined endpoint was the assessment of significant levels of pre-defined pollutants in the observed matrix such as honey bee and beehive products.

## 2. Methods

### 2.1. Study Design

The design of this study was a systematic literature review. We defined inclusion and exclusion criteria prior to the literature search. The risk of publication bias, meaning that only studies that report significant results are published, was considered. To minimize this bias, we carried out a thorough quality and validity assessment. We defined eligibility criteria and validity assessment prior to the literature search. We established a research protocol and registered each step of the literature search. Studies were included in this review based on various characteristics concerning study design and methodology. Only primary studies were considered. Moreover, the publication had to be peer-reviewed and published in English or German. To further control the quality of the publication, it had to be published in a journal with a rank of at least Q2 on the Scimago Journal & Country Rank [20]. The sampling method as well as the extraction method had to be described precisely, and the assessment of results had to be transparent and comprehensible. 

Eligible studies were subject to critical appraisal. Since there was no validated tool designed at the point of this research to assess bias in environmental studies, we assessed the risk of bias by examining the study design and methods to determine whether adequate steps were taken to protect against bias. Based on the style of the Cochrane Risk of Bias tool [21], we carried out a modified risk validity assessment. 

The validity of the studies was categorized as high, medium, low, or unclear. We defined the validity criteria in accordance with Bilotta et al. (2014) and based on a previous review by Macura et al. (2019) [22,23]. Studies defined as having high or medium validity were included in this systematic review, whereas those categorized as having low or unclear validity were excluded. Validity was assessed at an internal level, such as the susceptibility to bias or confounding, as well as at an external level, such as the relevance of the assessed study to address the review question. To assess validity at an external level, the included studies had to use honey bees or beehive products such as wax, honey, or pollen to assess environmental contamination. Moreover, to enable the evaluation of the sensitivity of honey bees to certain pollutants, the obtained results had to be compared with at least one site from a different landscape type or with a passive sampler (e.g., from soil, air, or water).

### 2.2. Search Strategy

To conduct the literature search and include relevant literature, we scanned the databases Medline, Scopus, and Cochrane Library. For the search in the online databases, we used the following keywords: (bees OR pollinators) AND (biomonitor OR bioindicator) AND (contamination OR pollution). The time frame was set from 2010 to 2020. We defined the inclusion criteria in regard to study design as well as to the publication suitability to address the defined research question. 

In the first screening process, we scanned titles and abstracts of the yielded articles for adherence to the eligibility criteria. If in this step, the publication did not show any clear signs of incoherence with the eligibility criteria, we searched the section “similar articles” (PubMed) or “related documents” (Scopus) to obtain further suitable articles. Duplicates were removed. After the sampling of potentially eligible articles, we screened the full text. The evaluation of full adherence to the eligibility criteria and the internal validity of the studies was carried out simultaneously. We excluded publications not meeting the eligibility criteria or being rated as having low or unclear validity and stated reasons in the research protocol. 

### 2.3. Literature Analysis

Concerning the study design, we aimed to reduce the risk of selection and sampling bias by establishing inclusion and exclusion criteria before initiating the search in the online databases and following the PRISMA guidelines throughout the reviewing process [24]. Moreover, the quality of each included study was thoroughly assessed. However, the significance of this review is compromised by the high heterogeneity of the included studies, which would not allow for a meaningful comparison. Nevertheless, this probability was considered before starting the writing process, which is why it was decided to follow a descriptive rather than an analytical approach and prioritize summarizing results rather than comparing values. We were not able to register this review on the International Prospective Register of Systematic Reviews (PROSPERO) due to the COVID-19-pandemic-related overload of the registration system at this time. Prior to starting the literature search for this review, we checked academic online sources to see whether a similar review already existed.

The retrieved studies differed regarding samples, methodology, and assessed contaminants and were, therefore, prone to variation. Due to this heterogeneity, we were not able to conduct a meta-analysis. Data extraction and the attention to potential heterogeneity between studies concerned the matrix or matrices of interest, the assessed contaminant(s), the methodology, the effect measures, the location (urban/industrial/rural areas), the number of samples, the timepoints of measurements, and the time and timespan of each study.

## 3. Results

### 3.1. Results of Systematic Literature Review

**Figure 1:**
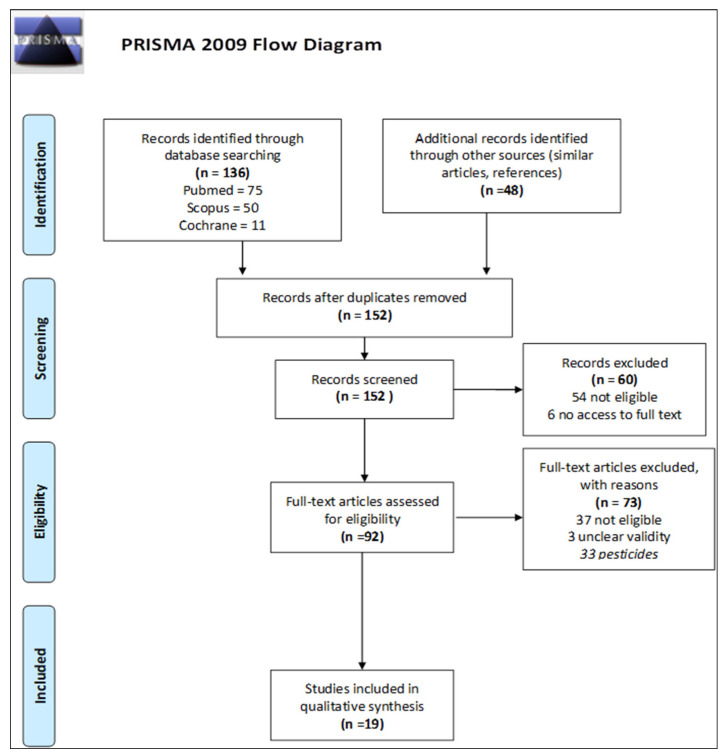
Prisma Flow Diagram: steps of the literature search and article screening process.

Figure 1 shows the different stages of the literature review integrated into a PRISMA Flow Diagram. In total, we screened 152 articles. The initial search in the databases PubMed, Cochrane, and Scopus generated 136 articles. Forty-eight articles were further added by scanning the references and “similar articles” section. PubMed was the first database we scanned, and it generated seventy-five results. The search in Scopus showed twenty-four articles that had already been a result of the PubMed search, and we found eight duplicates in the Cochrane database. Fifty-four articles were excluded after the first screening as they did not meet the predefined eligibility criteria. Six articles could not be further considered, as there was no access to the full texts [25,26,27,28,29,30]. These steps of identification and screening resulted in ninety-two articles, whose eligibility was then further reviewed. 

At the stage of full article screening, we excluded seventy-three studies. More than thirty of the studies found in the literature search addressed the evaluation of pesticide or veterinary drug residues in honey bees and hive products (*n* = 33). Over the last decade, methods for extracting pesticides and detecting concentrations of this group of chemicals in hive matrices have been developed, evaluated, and adapted specifically for this purpose [31]. Moreover, these studies focused on a rather exclusive group of pollutants, which often directly affect the beehive's health or lead to adverse behavioral effects. Therefore, they would have required attention that was beyond the scope of this systematic review and, thus, were not considered in the further analysis. 

The process of screening and validity assessment resulted in nineteen articles, which were included in this literature review (Table 1). Of those, sixteen were found to exhibit high validity characteristics. Three studies were ranked as medium validity. 

### 3.2. Characteristics of Reviewed Studies

We found a high variety of studied pollutant(s) of interest, matrices, and sampling in the nineteen included studies. Of the nineteen studies included, thirteen reviewed contaminant levels in a single hive matrix, as shown in Table 2 [10,14,34,35,36,37,39,40,41,44,46,47].

**Figure 2:**
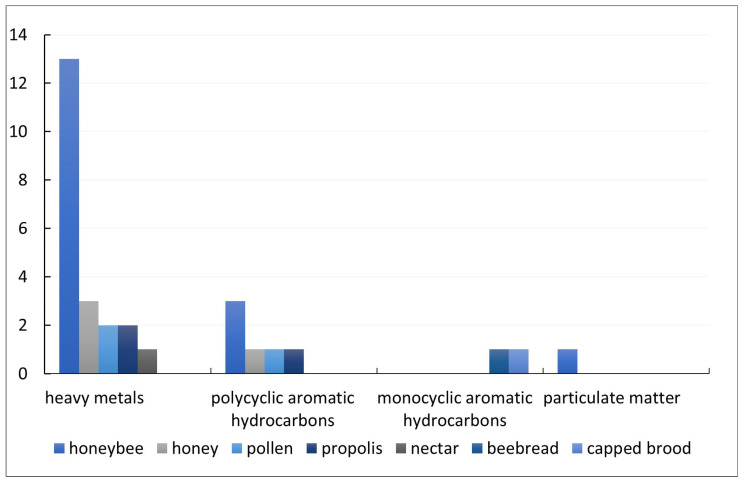
Types of pollution and matrices applied to assess these pollutants (in absolute numbers).

The maximum number of observed beehive matrices was three [16,32,33]. Honey bees (worker bees and dead honey bees combined) were the most frequently studied matrices (*n* = 17) [10,14,16,32,33,34,35,36,37,38,39,40,41,42,43,46,47]. Honey was the matrix of interest in four studies [16,32,33,36] and pollen, nectar, propolis, bee bread, and larvae were the least-observed matrices [16,32,33,38,42,44,45] (see Figure 2). Wax was never used as a matrix. Regarding the source of pollution, most of the included studies investigated heavy metal pollution (*n* = 15) [10,14,16,33,34,35,36,37,38,39,40,41,43,44,47], while four studies focused on air pollution [32,42,45,46]. 

### 3.3. Implications of Using Different Matrices

#### 3.3.1. Foraging Honey Bees

Foraging honey bees were the most frequently analyzed matrix, regardless of the contamination type of interest: Seventeen studies included in this literature review used honey bees as monitor matrices [10,14,16,32,33,34,35,36,37,38,39,40,41,42,43,46,47]. Alive honey bees were sampled directly from the hive or at the hive entrance or exit. A large proportion (*n* = 12/17, 70.6%) of the studies using honey bees as a matrix collected foraging honey bees when they returned to or exited from the hive [10,14,16,32,33,35,36,37,38,41,42,46]. In five studies, honey bees were collected from the outer frames of the beehive [34,39,40,43,47]. Although reasons for the different sampling procedures were not stated, sampling from the outer frame was only applied in studies focusing on pollution with heavy metals [34,39,40,43,47]. As smoking of the hive was usually applied to enable this sampling procedure, this procedure might alter the pollution levels in regard to PAHs [48]. Regardless of the sampling method, honey bees were immediately frozen after collection and then later in the laboratory, homogenized, and analyzed. 

Fifteen of the seventeen studies using honey bees as matrices analyzed contaminant levels solely in the whole honey bee [10,14,32,34,35,36,37,38,39,40,41,42,43,46,47]. Notably, the samples were not washed prior to analysis. More than 82% (*n* = 14/17) of the studies used honey bees as biomonitors for heavy metal (HM) pollution [10,14,16,33,35,36,37,38,39,40,41,43,47]. The overall objective was to evaluate the use of the honey bee matrix to assess for pollution by comparing levels in a potentially highly polluted area (e.g., an urban/industrial/agricultural site) with those detected at a proposedly uncontaminated control site (a wildlife/natural area). Herein, half of the studies reported strong spatial variations for at least some of the assessed HMs [16,34,37,39,40,41,43]. Moreover, eight of the fourteen studies on honey bees as biomonitors for HM exposure were conducted over a timeframe of several months or even years to draw conclusions about possible seasonal influences on pollution levels [10,16,32,34,37,40,43,47]. Approximately two-thirds of the studies assessing seasonal variations (*n*= 6/8, 66,7%) detected them [10,16,34,37,43,47]. For example, Zarić et al [43]. measured HM concentrations in honey bees over a study period of three years and reported increasing levels during this period. Each sample of a honey bee covers a timeframe of approximately three weeks prior to the sampling point [14]. This timeframe is explained by the fact that the worker bee leaves the hive and forages only in the last phase of its life [13,14]. Thus, a larger number of sampling dates covers a larger timeframe and consequently provides a broader picture of contamination in an area.

To monitor pollution with PAHs, three out of three included studies applied the honey bee as a biomonitor [32,42,46]. Of those, two studies assessed the influences of the landscape context [32,46]. Both reported that the evaluation of levels in the honey bee allowed them to assess the significant influence of the surrounding environment [32,46]. Moreover, Lambert et al. stated that honey bees can be used to depict contamination levels at the widest dispersion whilst also displaying peak contamination events [32]. Likewise, Kargar et al. found that honey bee samples allow the creation of a clearer fingerprint of the PAH profile than other biological matrices [42]. 

To enable more meaningful conclusions on the reliability of honey bees as biomonitors, Gutierrez et al [37]. compared the values obtained using honey bees with those detected with conventional physicochemical monitoring stations. The authors discovered that concentrations found in honey bees and those detected with physicochemical stations did coincide. Moreover, the monitoring of honey bees once revealed even higher levels than those detected in mobile stations [34]. This is in accordance with the findings of Porrini et al., who stated that the analysis of the whole honey bee body allows the consideration of both internal and external contamination levels [5]. To assess for correlations and potential mutual contamination pathways, Zarić et al. [43] took soil samples in addition to the whole honey bee. The authors did not detect significant correlations between concentrations in soil and honey bees, which was expected due to the honey bees’ own bioconcentration of elements. Likewise, Ilijević et al. [47] sampled atmospheric particulate matter (PM) by placing buckets next to the tested apiaries. This allowed for distinguishing whether the elements accumulated in the bees originated from air pollution or from uptake from nectar, pollen, or water. 

Honey bees from the same site may differ in their foraging activity and, therefore, in their contamination uptake. Of note, Zieba at al. [45] propose to monitor at least three honey bee colonies per site to create a meaningful mean value. This recommendation resulted from comparing the coefficients of variance of beebread and capped brood samples from different colonies at each site and finding a wide variance. In contrast, Cochard et al. [46] examined the variance between honey bee samples from hives in the same location to test for between-hive effects and found no significant difference. However, most of the included studies assessed at least two hives per site (*n* = 16/19, 84%). Furthermore, it is important that the sampled apiaries are situated far enough apart from each other when evaluating spatial variation between two or more sites. Satta et al. [16] conducted a discriminant analysis, revealing that honey bee analysis achieved a discrimination accuracy rate of 72%, whereas soil analysis demonstrated a higher accuracy rate at 83%. This lower discriminative power was most likely caused by the relatively short distance between hives of approximately 7 km, which might have led to an overlap of foraging areas.

#### 3.3.2. Honey

Four of the included studies assessed the levels of pollutants in honey collected directly from the hive by cutting out fresh combs [16,32,33,36]. Contamination in honey is believed to be caused either by pollutants in nectar and pollen or by the honey bee itself [32]. Satta et al. investigated the levels of the three HMs, cadmium, chromium, and lead, in honey, pollen, and honey bees from apiaries at post-mining sites [16]. While they found a significant difference in the cadmium content of honey, which was higher in the prior mining areas than in the control area, this was not the case for the other metals. In addition, the HM levels found in honey were generally low. In addition to beehive samples, the researchers took samples from stream water and soil in proximity to the beehives. The subsequent regression analysis showed that there were more significant relationships between the soil and water samples and the honey bees and pollen (*n* = 5) than with honey (*n* = 1).

In two studies, Lambert et al. used honey bees, pollen, and honey from hives in the same areas in France for lead [33] and PAH content assessment [32]. Regardless of the contaminant of interest, both studies found the lowest average mass fraction (referred to as concentration hereafter) in honey (Pb in honey: 0.05 µg/g, bees: 0.22 µg/g, and pollen: 0.24 µg/g; PAH in honey: 0.82 µg/kg, bees: 7.03 µg/kg, and pollen: 7.1 µg/kg). Moreover, lead concentrations in honey samples were below the limit of detection in 30% of cases and ranged between the limits of detection and the limit of quantification in 10% of cases. Furthermore, Lambert et al. [32] could not find any evidence of PAH transmission from honey bees to honey or from pollen to honey.

In accordance with these results, Ruschioni et al. [36] found a similar pattern. When comparing levels of chromium (Cr) and nickel (Ni) in honey bees and honey, they found less seasonal variation and lower levels in honey (monthly exceedance of threshold: Cr—honey bees 20% and honey 11%; N—honey bees 5% and honey 2%). The authors concluded that the high HM levels found in honey might correspond to periods of continuously high pollution, allowing for the pollutants to accumulate in the honey. These results support the findings of other studies that honey is not a good biomonitor for heavy metal pollution [13,17,49]. As for PAH pollution, so far, there is only one study using honey to assess this type of pollution [32]. While it found honey to provide discrimination between polluted and non-polluted sites, in comparison with honey bees and pollen, honey had the lowest concentrations [32].

The reason for the generally low usefulness of honey as a biomonitor potentially lies in the honey bee’s mechanism to block certain pollutants as well as the physicochemical composition of honey. Honey bees are known to produce relatively clean honey, one filter mechanism being the physical barrier of the stylets of the mouthpart, which does not allow particles as large as or larger than 100 µm to be ingested [50]. Dzugan et al. studied the transference of toxic elements from honey bees to honey by calculating transfer coefficients [51]. They found the honey bee body to successfully prevent the transference of two of the elements studied (i.e., Cd and Tl) [51]. Apart from the physical filtering ability of honey bees, the elemental composition of honey strongly depends on its botanical origin, as the uptake from soil to plant is plant-species-specific and the atmospheric uptake of pollutants depends, e.g., on the morphology of the plant [16,32,51,52,53,54,55]. This difference might lead to levels being influenced by the natural composition of the sampled honey. To account for this possible influence by the flower species, Satta et al. [16] performed a melissopalynological analysis, which confirmed the variety of flowers in the sample areas. As a result, identifying plant species in an area that are more sensitive to pollution could lead to an increased reliability of the honey as a biomonitor.

#### 3.3.3. Pollen

Nectar, honey, and pollen can represent both the uptake of pollutants from the soil into the plant and the deposition of pollutants into the atmosphere [16,32]. Pollen is thought to accumulate greater amounts of pollutants due to its lipophilicity and high viscosity [45]. Moreover, pollen usually derives from numerous flowers [16]. Hence, the sampling of pollen offers a wider range of sampling sites [5,16]. A common method of collecting pollen is to place pollen traps at the entrance of the hive three days to a week before sampling [16,32,33]. 

Pollen was the matrix of interest in three studies included in this review [16,32,33]. Heavy metals were the pollutants of interest in two studies [16,33], whereas levels of PAHs were assessed in one study [32]. All three studies reported significant spatial variations in pollutant levels in pollen [16,32,33]. Furthermore, Satta et al. found a strong relationship between the lead content in soil and in pollen [16].

Lambert et al. reported similar levels of lead in honey bees and pollen [33]. Furthermore, the analysis of pollen allowed the observation of seasonal variations during the study period [33]. Lambert et al. are the only authors to date who have used pollen to monitor PAH concentrations [32]. Although their pollen samples effectively detected contamination spikes, they encountered limitations in their study, primarily stemming from a notably small sample size due to adverse weather conditions. Consequently, they were unable to construct a generalized linear model. Also, their research did not uncover any evidence of PAH transfer from pollen to honey bees.

#### 3.3.4. Propolis

Propolis is a sticky, resinous substance that is produced by worker bees as a product of plant resin, wax, and salivary secretion [38,56]. Worker bees mix the collected resin with beeswax, enzymes, and other substances, creating a malleable and adhesive substance. Bees use propolis for several important purposes within the hive, such as filling cracks in the hive walls and strengthening the structural integrity of the hive. Outside the beehive, humans have used propolis for centuries in various forms, like tinctures, capsules, or ointments, in the pharmaceutical and cosmetic industry due to its anti-inflammatory and antioxidant effects. So far, there are few studies that have assessed pollution in propolis, most of them focusing on the direct uptake of pollution present in propolis via its application in food products [38,57,58,59]. Its application as a biomonitor has been rare thus far, even though propolis accumulates pollutants from air, water, and soil [13]. However, the proportion of propolis collectors in a hive is low. When monitoring HMs, trace element concentrations differ depending on the geographical region as well as on the plant species from which the resin was collected [38,57].

Among the studies included in this literature review, only the works by Matin et al. and Kargar et al. utilized propolis as a means to evaluate environmental pollution levels [38,42]. Their first study focused on the concentrations of HMs in honey bees, propolis, and pine tree leaves [38]. Interestingly, they found the highest concentrations in propolis in comparison with the other matrices. This finding was followed by their second study, in which they used the same matrices to assess pollution with PAHs [42]. Propolis was again found to show the highest concentrations of all sampled matrices. The authors further conducted a PCA to create a source profile of the detected PAHs in propolis. Three out of four components were attributed to emissions from cars and pyrogenic processes, whereas one component was attributed, rather, to the environmental degradation in the study area. The study found an association between three-ringed PAHs, such as phenanthrene, with four-ringed PAHs, such as benzo(a)pyrene. These findings suggested creosote as a source, which is potentially due to a lack of natural resources for propolis production, causing honey bees to use gummy substances like tar and asphalt as a substitute for resin [38,42].

#### 3.3.5. Beebread

Few studies have used beebread to assess levels of pesticides [60] or insecticides [61]. In this review, only one study conducted by Zieba et al. was included that used this matrix to assess levels of monocyclic aromatic hydrocarbons (MAHs) [45]. Beebread derives from pollen, which is further processed by the honey bee and stored in the hive to serve as a nutritional source. Beebread was collected by cutting out a piece of comb from the hive and later analyzed using GC/MS. The authors reported a significant variation in the assessed levels of MAHs between the two years of sampling, but no seasonal variation between spring and summer samples. Furthermore, there were no significant variations concerning the study sites (urban and industrial areas). Toluene was found at the highest concentration in beebread and the additionally sampled capped brood. Additionally, during the summer, it significantly exceeded beebread levels compared with spring.

#### 3.3.6. Capped Brood and Nectar

Worker honey bees usually live for approximately forty-five days, passing different development stages and roles in the beehive [13]. Zieba et al. [45] hypothesized that contamination levels in honey bees at the end of their larval development stage could be higher than in adult forager bees. They attributed this potential difference to a change in the main food source, with capped brood feeding mainly on pollen and foraging bees on nectar, which is usually less polluted. During most of its life, the worker bee is exposed to the pollutants in the food it eats and the pollutants carried into the hive by the collecting bees when depositing pollen and nectar. This means that monitoring bee larvae or nursing bees could provide a more comprehensive picture of the exposure detected in the hive and the contaminant content in pollen and nectar. However, the study found generally low levels of pollution in capped brood compared with simultaneously sampled beebread. Furthermore, the application of the capped brood did not display any significant temporal or spatial variations.

Only one study using nectar as a biomonitor was included in this literature review [37]. Nectar can be contaminated with pollutants from the air and via the uptake of pollutants from the soil [37,44]. However, nectar has been shown to be the least-contaminated beehive product [16,44]. Firstly, due to the structure of the flower, it is relatively well protected from pollutants from the air [45]. Secondly, it is strongly influenced by weather conditions. During heavy rain, nectar is washed out and at high temperatures, it evaporates from the flower. This leads to its constant renewal so that lower pollution levels are observed when this matrix is used as a biomonitor [14,45]. In addition, the degree of nectar contamination depends on the season, with nectar flow being higher in spring than in summer and fall [5]. 

Regarding airborne pollution, nectar is considered a relatively unsuitable biomonitor because of its low-fat content and low bioaccumulation potential [32,62]. Although nectar has a lower degree of contamination and is less suitable to represent seasonal and spatial variation compared with the honey bee matrix, Gutierrez et al. [44] noted that it could be useful as a complementary matrix. The monitoring of nectar revealed peak levels of certain pollutants, which were missed by physicochemical stations monitoring air pollution around the study area. Moreover, strong correlations were found between concentrations in nectar and those reported by the physicochemical stations, displaying a relationship between concentrations in nectar and in air.

## 4. Discussion

In recent times, there has been a notable surge in the popularity of the One Health concept, which underscores the interdependence of environmental, animal, and human health [18]. Within this context, the utilization of natural organisms capable of accumulating contaminants in their tissues over time, commonly referred to as biomonitors, has gained prominence. Among these biomonitors, honey bees stand out as especially valuable tools, adept at accumulating pollutants from the air, soil, and water within a defined radius during their foraging expeditions [10,14,34,35,36,37,39,40,41,44,46,47]. This systematic literature review provided a comprehensive overview of the utilization of the honey bee as a tool for pollutant monitoring, encompassing articles published within the decade spanning from 2010 to 2020. In total, we reviewed nineteen studies to synthesize the state of research in this area and the reported approaches, methods, and applications when using beehive products as biomonitors.

The honey bee has a remarkable capacity to amalgamate pollution data over a defined geographical area and is relatively easy to sample. Additionally, noteworthy results have also been obtained via the analysis of pollen, which presents the advantage of being easily collectible from various locations [16,32,33]. However, the accumulative potential of pollen is highly dependent on its lipophilicity and, therefore, on its plant species [32,63]. Moreover, pollen might be more sensitive to changing weather conditions, as low temperatures limit honey bees’ foraging activity and, therefore, the sampling of pollen [32]. As for honey, Satta et al. [16] found that it could depict seasonal as well as spatial variations for some heavy metals. However, all studies assessing levels of pollution in honey in comparison with the whole honey bee found that its reliability was rather low [16,32,33,36]. In regard to propolis, nectar, beebread, and capped brood, the number of studies using these matrices for biomonitoring purposes is currently too small to derive a meaningful conclusion.

The reviewed literature shows that the limits of reliability of beehive matrices relate to sampling seasons, weather conditions, and foraging activity. Depending on the matrix used as well as on the contamination type of interest, the reliability and usefulness of beehive matrices vary. This is exemplified by pollution with PAHs and the typical sampling seasons of beehive matrices and foraging honey bees in particular. PAH peak levels are usually found in winter, which is explained by the higher emission of certain PAHs in colder months due to an increase in heating activity and vehicular traffic [64] and by the sensitivity of some PAHs to photodegradation [65]. However, foraging activity naturally decreases in winter months [13], leading to less exposure of honey bees to these pollutants. Moreover, most of the included studies limited sampling to seasons with high foraging activity. Since the assessment of critical PAH levels would require a consistent sampling matrix throughout the year, a natural “wrong place, wrong time” dilemma occurs, which leads to the limited reliability of the honey bee as a single biomonitor for PAH pollution.

Regardless of the type of pollution, higher levels were usually found in summer. Nonetheless, Ilijević et al. [32] reported peak levels of cadmium and chromium in honey bees in winter. Since winter bees rarely leave the hive and, therefore, are less exposed to atmospheric pollutants, this finding can be most likely ascribed to the food that is consumed in winter. Based on this observation, a change in the monitored beehive matrix in winter to those better depicting the contamination of food sources seems favorable. However, to date, there is only one study focusing on the assessment of pollutants in matrices like beebread and capped brood [45], which therefore minimizes their reliability. As for other food sources like pollen and propolis, few studies have assessed the levels of pollutants in these matrices [16,32,33,38,42].

Foraging activity is not only influenced by temperature but also by wind speed, humidity, and cloud cover [5]. In addition to these natural weather conditions, environmental factors such as poor air quality or deforestation and urbanization alter honey bees’ foraging [5,66]. Cho et al. [66] found that foraging duration increases proportionally to increased PM concentrations. Moreover, if food sources are scarce, honey bees increase their usual foraging distance of a few hundred meters around the hive to up to kilometers in search of food [5]. This might lead to unexpectedly low concentrations of pollutants in honey bees situated at suspected polluted sites. However, surprisingly high levels of pollutants in areas far from pollution sources might be explained by drift and long-range transport from distant pollution sources [36,47]. These results may indicate that sampling sites should not be selected a priori but should instead be chosen to represent area-wide concentrations [39].

Using the One Health approach, a safer world for all living creatures, fostering a balance between human development and the preservation of ecosystems, can be achieved [18]. This concept not only protects human health but also recognizes the intrinsic value of biodiversity and the critical role animals and the environment play in our shared well-being. From an environmental health viewpoint, the honey bee can be considered as a model organism for the One Health concept [19]. Its application as a biomonitor allows for the attainment of relevant levels of pollutants as well as for drawing conclusions on the qualitative state of the surrounding environment. The assessment of the whole honey bee body enables the integration of pollutants present in the ambient air, soil, and water and therefore delivers an overall picture of the state of pollution in a specified area. As an organism that itself is strongly influenced by weather conditions, changing landscapes, and pollution [42,44,66], the biomonitoring of the honey bee delivers valuable information on the state of the shared environment. An outstanding example of this is the findings of Kargar et al. [42], who reported that due to diminishing natural resources, the studied honey bees used tar and asphalt for propolis production. The threat to overall human health that comes with this degradation of natural ecosystems is clear.

### Limitations and Perspectives for Further Research

Sampling the whole honey bee produced the most reliable results; however, the number of studies using this matrix is significantly higher than those analyzing other beehive matrices. Therefore, a potential positive shift of results toward the reliability of the honey bee can be assumed, whilst a meaningful statement about other beehive matrices cannot be made at this point. There is a clear lack of studies focusing on the possibilities of using beehive matrices as biomonitors throughout the whole year, with studies mostly taking place during the active foraging period of the honey bee. The number of studies assessing levels of pollutants in beehive matrices in winter is rare, and there is room in the research regarding alternative monitoring matrices and the usage of matrices like beebread. Furthermore, the application of a complementary matrix to outweigh the limitations of the honey bee has been proposed, but there are no studies that have followed this approach yet.

In regard to pollution sources, the majority of included studies focused on pollution with heavy metals, which represents a rather long-term, accumulating pollution form. Studies on newer pollutants such as rare earths are missing. Furthermore, in pollution monitoring, the interpretation of the levels achieved in terms of reference thresholds or levels of concern is essential. However, reference values for various pollutants are often explicitly defined for soil, water, or air pollution. Since honey bees can accumulate pollutants via multiple pathways, reference values are often lacking. Additionally, it is crucial to consider the heavy metal load when interpreting the determined values, as certain heavy metals may naturally occur at higher concentrations at specific sites [5]. Porrini et al. [5] therefore suggest using values from a control site that is relatively uncontaminated and similar to the test site to account for natural factors that influence the contamination pattern.

This literature review focused solely on the usage of honey bees of the species *Apis mellifera* as biomonitors whilst excluding other pollinators such as wild honey bees or bumblebees. The inclusion of more pollinators in a future review would enable a more thorough, inclusive picture.

The honey bee itself has proven to be the most reliable matrix, allowing the detection of spatial and seasonal variations in certain pollutants. However, the reliability of honey bees or beehive products as biomonitors is influenced by numerous variables. Some of them are rather uncertain, whereas others can be factored in by following certain recommendations. First, a minimum of three beehives per site should be monitored to account for variations in the same colony. Second, to test for spatial variation, the different sites should be distanced at least 8 km from each other to minimize the chance of areas overlapping due to the flight radius of the foraging honey bees. Third, depending on the pollutant of interest, complementary beehive matrices or other monitoring methods could be needed to achieve more meaningful results. Moreover, study authors should be aware that honey bees’ foraging is influenced by numerous factors, which makes the creation of standard values and methods difficult.

## 5. Conclusions

Using honey bees as biomonitors presents a promising avenue for continuous pollution monitoring, complementing traditional fixed monitoring stations. This approach offers a more comprehensive and intricate perspective of environmental health, aligning with the One Health concept, which recognizes the interconnectedness of environmental, animal, and human health. Via honey bee monitoring, we can gain valuable insights into the environmental conditions and, by extension, the well-being of communities residing in these environments. Nevertheless, the current body of research in this field is limited, often focusing on contrasting landscapes and assessing similar types of pollutants without sufficient contextualization. This highlights the need for further investigation and standardization in honey bee biomonitoring studies. Such efforts will not only enhance our understanding of environmental health but also contribute to the development of accessible and widely applicable monitoring practices. From an environmental health perspective, standardized studies are needed to possibly create an accessible monitoring protocol as well as to enable a more homogenous interpretation of levels found in the beehive matrices.

## Figures and Tables

**Table 1:** Articles included in this review (*n* = 19, ^a^ and ^b^ refer to two different papers by the same authors) in chronological order.

Authors, Publication Year, Reference	Article Title	Journal
Perugini et al., 2011 [10]	Heavy Metal (Hg, Cr, Cd, and Pb) Contamination in Urban Areas and Wildlife Reserves: Honey bees as Bioindicators	*Biological Trace Element Research*
Lambert et al., 2012 [32] ^a^	Bees, Honey and Pollen as Sentinels for Lead Environmental Contamination	*Environmental Pollution*
Lambert et al., 2012 [33] ^b^	Polycyclic Aromatic Hydrocarbons: Bees, Honey and Pollen as Sentinels for Environmental Chemical Contaminants	*Chemosphere*
Satta et al., 2012 [16]	Combination of Beehive Matrices Analysis and Ant Biodiversity to Study Heavy Metal Pollution Impact in a Post-Mining Area (Sardinia, Italy)	*Environmental Science and Pollution Research*
van der Steen et al., 2012 [34]	Spatial and Temporal Variation of Metal Concentrations in Adult Honey Bees (*Apis mellifera* L.)	*Environmental Monitoring and Assessment*
Badiou-Beneteau et al., 2013 [35]	Honey Bee Biomarkers as Promising Tools to Monitor Environmental Quality	*Environment International*
Ruschioni et al., 2013 [36]	Biomonitoring with Honey bees of Heavy Metals and Pesticides in Nature Reserves of the Marche Region (Italy)	*Biological Trace Element Research*
Gutierrez et al., 2015 [37]	Assessment of Heavy Metal Pollution in Cordoba (Spain) by Biomonitoring Foraging Honey Bee	*Environmental Monitoring and Assessment*
Negri et al., 2015 [14]	Honey Bees (*Apis mellifera*, L.) as Active Samplers of Airborne Particulate Matter	*PLOS One*
Matin et al., 2016 [38]	Bio-Monitoring of Cadmium, Lead, Arsenic and Mercury in Industrial Districts of Izmir, Turkey by Using Honey Bees, Propolis and Pine Tree Leaves	*Ecological Engineering*
van der Steen et al., 2016 [39]	Think Regionally, Act Locally: Metals in Honey Bee Workers in The Netherlands (Surveillance Study 2008)	*Environmental Monitoring and Assessment*
Zarić et al., 2016 [40]	Metal Concentrations Around Thermal Power Plants, Rural and Urban Areas Using Honey Bees (*Apis mellifera* L.) as Bioindicators	*International Journal of Environmental Science and Technology*
Giglio et al., 2017 [41]	*Apis mellifera* ligustica, Spinola 1806 as Bioindicator for Detecting Environmental Contamination: A Preliminary Study of Heavy Metal Pollution in Trieste, Italy	*Environmental Science and Pollution Research*
Kargar et al., 2017 [42]	Biomonitoring, Status and Source Risk Assessment of Polycyclic Aromatic Hydrocarbons (Pahs) Using Honey Bees, Pine Tree Leaves, and Propolis	*Chemosphere*
Zarić et al., 2017 [43]	Use of Honey Bees (*Apis mellifera* L.) as Bioindicators for Assessment and Source Appointment of Metal Pollution	*Environmental Science and Pollution Research*
Gutierrez et al., 2020 [44]	Assessing Heavy Metal Pollution by Biomonitoring Honey Bee Nectar in Cordoba (Spain)	*Environmental Science and Pollution Research*
Zieba et al., 2020 [45]	Usefulness of Bee Bread and Capped Brood for the Assessment of Monocyclic Aromatic Hydrocarbon Levels in the Environment	*Environmental Pollution*
Cochard et al., 2021 [46]	PAH7 Concentration Reflects Anthropization: A Study Using Environmental Biomonitoring with Honey Bees	*Science of the Total Environment*
Ilijević et al., 2021 [47]	Anthropogenic Influence on Seasonal and Spatial Variation in Bioelements and Non-Essential Elements in Honey Bees and their Hemolymph	*Comparative Biochemistry and Physiology C-Toxicology and Pharmacology*

**Table 2:** Characteristics of the reviewed studies (*n* = 19, ^a^ and ^b^ refer to two different papers by the same authors) in alphabetical order.

Study	Pollutant(s)	Matrices	Analytical Technique	Sampling (*n*)		
				Collection	Locations	Hives
Badiou-Beneteau et al., 2013 [35]	HM	Honey bees	ICP-MS	4	2	6
Cochard et al., 2021 [32]	PAH	Honey bees	GC-MS/MS	12	36	108
Giglio et al., 2017 [41]	HM	Honey bees	ICP-MS	1	2	2
Gutierrez et al.2015 [37]	HM	Honey bees	ICP-OES	20	5	10
Gutierrez et al., 2020 [44]	HM	Nectar	AAS	20	5	10
Ilijević et al., 2021 [47]	HM	Honey bees (bucket collection)	ICP-OES+/−ICP-MS	3	3	15
Kargar et al., 2017 [42]	PAH	Honey bees Propolis (pine tree leaves)	GC	1	5	5
Lambert et al., 2012 ^a^ [32]	HM	Honey bees Honey Pollen	AAS	8	18	144
Lambert et al., 2012 ^b^ [33]	PAH	Honey bees Honey Pollen	GC/MS	8	6	48
Matin et al., 2016 [38]	HM	Honey bees Propolis (pine tree leaves)	EAS/FAS	1	5	5
Negri et al., 2015 [14]	PM; HM	Honey bees (sediment)	SEM-EDX	1	4	11
Perugini et al., 2011 [10]	HM	Honey bees	AAS	6	8	24
Ruschioni et al., 2013 [36]	HM	Honey bees Honey	ICP-AES/GC/MS	15	11	22
Satta et al., 2012 [16]	HM	Honey bees Honey Pollen Stream water Soil Ants	EAS	12	3	9
van der Steen et al., 2016 [39]	HM	Honey bees	ICP-MS	1	9	750
van der Steen et al., 2012 [34]	HM	Honey bees	ICP-AES	6	3	9
Zarić et al., 2017 [43]	HM	Honey bees (soil)	ICP-OES	5	4	10
Zarić et al., 2016 [40]	HM	Honey bees	ICP-OES	2	6	13
Zieba et al. 2020 [45]	BTEX	Bee bread Capped brood	GC-MS	4	4	12

Note: BTEX = benzene, toluene, ethylbenzene, and xylenes, HM = heavy metal, PAH = polycyclic aromatic hydrocarbon, PM = particulate matter, *n* = number, ICP-MS = inductively coupled plasma—mass spectrometry, ICP-OES = inductively coupled plasma—optical emission spectroscopy, GC-MS = gas chromatography—mass spectrometry, EAS = electron absorption spectroscopy, FAS = flame absorption spectroscopy, and AAS = atomic absorption spectroscopy.

## Data Availability

No new primary data were created during this literature review.
